# Results from a systematic programme of evaluating COVID-19 reinfection cases in the early phase of the pandemic, Singapore

**DOI:** 10.1186/s12879-023-08056-8

**Published:** 2023-02-14

**Authors:** Glorijoy Shi En Tan, Christine Qiuhan Gao, Jievanda Shu Ying Ow, Thuan Thong Tan, Say Tat Ooi, Cui Lin, Raymond Tzer Pin Lin, Vernon Jian Ming Lee, Monica Chan, Yee Sin Leo, Shawn Vasoo

**Affiliations:** 1 Department of Infectious Diseases, Tan Tock Seng Hospital, Singapore, Singapore; 2 National Centre for Infectious Diseases, Singapore, Singapore; 3 Department of Infectious Diseases, Singapore General Hospital, Singapore, Singapore; 4 Division of Infectious Disease, Department of General Medicine, Khoo Teck Puat Hospital, Singapore, Singapore; 5 Ministry of Health, Singapore, Singapore; 6 Lee Kong Chian School of Medicine, Nanyang Technological University, Singapore, Singapore; 7 Level 3 Clinical Staff Office, Centre for Healthcare Innovation, 18 Jalan Tan Tock Seng, 308443 Singapore, Singapore

**Keywords:** COVID-19, SARS-CoV-2, Reinfection

## Abstract

**Objectives:**

The objectives of this study were to describe the coronavirus disease caused by SARS-CoV-2 (COVID-19) reinfection evaluation algorithm used in the early phase of the pandemic in Singapore and analyze the clinical and laboratory characteristics of the cases evaluated.

**Methods:**

We performed a retrospective case-control analysis including all COVID-19 cases evaluated for possible reinfection under the local COVID-19 reinfection evaluation programme between 1 June 2020-30 June 2021. Whole genome sequencing (WGS) was used as confirmatory testing. We compared all reinfection (“RI”) cases against those who were evaluated but eventually assessed not to be reinfection (“non-RI”).

**Results:**

There were 74 possible reinfection cases evaluated through the programme, of which 32 were subsequently classified as RI. There was strong statistical evidence that RI cases had a longer interval between 1st and 2nd episode (mean 297 days; 95%-confidence interval (CI) 267–327) compared to non-RI cases (mean 186 days; 95%-CI 144–228). The cycle threshold (Ct) value of initial polymerase chain rection (PCR) at 2nd episode was also found to be significantly lower in RI cases (mean 23; 95%-CI 20–26) compared to non-RI cases (mean 34; 95%-CI 32–36). There was no significant difference in the proportion of individuals who had fever, acute respiratory symptoms or asymptomatic in both groups. Delta and beta variants were most commonly identified from WGS and provide indication of re-infection as these were not ‘wild-type’ and were not circulating during the time period of the index infection.

**Conclusions:**

Using a combination of serologic, microbiologic and genomic criteria to evaluate possible reinfection cases is useful and can provide a framework for evaluation that may be modified for future similar situations.

## Introduction

Since the first case of coronavirus disease caused by SARS-CoV-2 (COVID-19) reinfection was reported in August 2020 [1], it has been recognized to be an entity with several similar reports confirming reinfection through molecular sequencing [2]. Over the course of the pandemic, increased transmission rates and variable testing capabilities have made the need to accurately diagnose reinfection all the more relevant to the pandemic response.

The development of new SARS-CoV-2 variants have risen concerns of increased transmissibility and potential immune escape. The degree to which individuals previously infected are immune to reinfections pose a challenge to public health professionals in case reporting. The diagnosis of reinfection is further complicated by a variable duration of viral shedding in individuals, in which viral ribonucleic acid is detected in respiratory specimens even after resolution of the acute illness, and is recognized to last for as long as months after the primary infection [3, 4].

This has led to revisions in case definitions globally for uniformity in case reporting. The United Kingdom Health Security Agency revised the definition in January 2022 and now considers an infection “a possible reinfection” if it took place at least three months after a previous confirmed infection, but does not confirm this through genetic sequencing [5]. United States Centers for Disease Control and Prevention uses a similar case definition, but also caveats circumstances in which severely immunocompromised persons can continue to shed SARS-CoV-2 detected by highly sensitive molecular amplification methods even more than 90 days despite being the same primary infection [6]. A number of studies have tried to characterize definitions for reinfection, distinguishing it from relapse and re-positivity [7, 8].

Since the beginning of the pandemic, Singapore adopted a national strategy to tackling COVID-19, with initial responses centred around border and movement restrictions, regular testing with stringent contact-tracing efforts and quarantine policies, amongst others [9]. Following large outbreaks of infection amongst migrant workers living in dormitories in 2020 [10], a surveillance method of “rostered routine testing” (RRT) for COVID-19 amongst key at-risk work sectors was implemented to ensure early detection of asymptomatic infection and facilitate prompt infection control measures[11].

In 2020, during the early phases of the pandemic where there was paucity of information on reinfection and understanding of its clinical implications, assessment of possible reinfection was largely assumed based on similar virus antigenicity. Following a surge of cases attributed to the Delta variant in the following year, continued RRT often detected polymerase chain reaction (PCR) positives in persons with a history of COVID-19 infection, prompting the development of an algorithm to evaluate cases for suspected COVID-19 reinfection through use of clinical features, epidemiologic and serologic data, cycle threshold (Ct) values from molecular amplification methods and genetic sequencing to determine reinfection. This systematic programme of assessing reinfection cases was key to understanding the true magnitude of each wave and the downstream clinical outcomes observed in reinfection cases.

### Aims and objectives

In this report, we aim to describe the COVID-19 reinfection evaluation algorithm used in the early phase of the COVID-19 pandemic in Singapore, in 2020, and analyze the clinical and laboratory characteristics of the cases evaluated in this programme, including confirmation with whole-genome sequencing (WGS) where available.

## Methods

### COVID-19 reinfection evaluation programme

At the start of the programme, a more cautionary stance was adopted where persons who had documented previous positive PCR or positive serologic tests unrelated to vaccination and subsequently tested PCR positive with any Ct value were admitted to a hospital isolation ward for further assessment. This was later revised to only include positive PCR with Ct values < 30 in May 2021. These patients may have been detected by RRT or tested due to clinical symptoms according to prevailing COVID-19 suspect criteria. All such cases were notifiable to the Ministry of Health Singapore (MOH).

These persons were assessed clinically with medical history and evaluated for compatible COVID-19 symptoms. Serological tests (the Elecsys Anti-SARS-CoV-2 S assay [Roche Diagnostics International Ltd., Rotkreuz, Switzerland] and the cPass SARS-CoV-2 Neutralization Antibody Detection Assay [GenScript, Singapore]) and bilateral nasopharyngeal swabs for SARS-CoV-2 PCR (GeneXpert SARS-CoV-2, Cepheid, Sunnyvale, California, USA) were taken on admission, and repeated after 24 h. All microbiologic tests were performed at a centralized National Public Health Laboratory (NPHL) to ensure consistency in reporting. NPHL then performed whole-genome sequencing (WGS) on all positive PCR tests where Ct values fell below 30.

The results from the above tests were consolidated and analyzed using an algorithm as shown in Table 1 for attending physicians to determine a preliminary assessment. This preliminary assessment was subsequently submitted to an expert panel comprising Infectious Diseases physicians, laboratorians and public health practitioners from MOH as the final arbiters to obtain concurrence before deciding on the final disposition and downstream public health actions necessary. Table 1 shows the five most common possible outcomes (non-exhaustive), and any deviation from the combinations shown may be escalated to the expert panel for further deliberation.

**Table 1:** Interpretation matrix to assess suspected reinfections

S/N	Result from Repeated PCR	Serology Result	Interpretation	Clinical actions to be taken	Action to be taken by Microbiology
1	Ct ≥ 30 or Repeat negative	Positive	Unlikely reinfection, likely persistent shedder	Consider discharging If there are any residual concerns, follow up in clinic with intervening medical leave and consider repeating tests	Nil
2	Ct ≥ 30 or Repeat negative	Negative	Unlikely reinfection, likely persistent shedder Possible waned immunity	Consider discharging. If there are any residual concerns, follow up in clinic with intervening medical leave and repeating tests	Nil
3	Ct < 30 or current Ct value less than Ct from last PCR test (if the same assay and sample type used)	Low quantitative serology titre (Anti-S)	Possible Reinfection, repeat serology (~ 2 weeks later)	If specimen error has been ruled out, manage as per protocols for suspect/confirmed case	Repeat PCR Consider viral cultures and sequencing Consider multiplex respiratory virus PCR to evaluate for other infections if symptomatic
4	Ct < 30 consistently on repeated testing or current Ct value significantly less than Ct from last PCR test (if the same assay and sample type used)	High quantitative serology titre (Anti-S)	Likely Reinfection		

All cases were eventually classified as confirmed reinfection (“RI”) or non-reinfection (“non-RI”).

### Analysis of cases evaluated under the programme

We retrospectively analyzed all cases evaluated under the programme between 1 June 2020 and 30 June 2021, as classified by an expert panel based on criterion outlined in Table 1. While microbiologic and serology criteria was used for evaluation of reinfection (Table 1), WGS was used as confirmation of reinfection where available. We thus performed a case-control analysis, comparing RI cases against non-RI cases, as determined by the programme, to evaluate the differences in clinical, radiologic and microbiologic features between the two populations. This will allow us to better understand these characteristics which may be useful to distinguish between true RI versus non-RI cases in the absence of routine WGS information.

Demographic, epidemiologic and limited clinical information (age, gender, date of first and suspected 2nd COVID-19 infection, clinical symptoms, chest radiograph results, Ct value of sequential PCR tests, vaccination history, WGS testing and serology tests results) were obtained from medical records. Preliminary and final evaluation made by attending physicians and the Expert Panel were obtained from records of panel deliberations.

### Statistical analyses

Epidemiological, microbiologic and clinical characteristics were compared between the cases and controls. An unpaired T-test or Mann-Whitney U-test was used to compare the difference in means for continuous variables, while a chi-square test or Fisher’s exact test was used to compare the difference in proportions for categorical variables.

All statistical analyses were performed using STATA 16.0 (StataCorp LLC, College Station, TX).

## Results

There were 74 reports of possible reinfection cases evaluated through the programme, of which 71 (96%) were male of median age 34 years (range 22–51)Thirty two were subsequently classified as RI cases while the remaining 42 were classified as non-RI cases. The reason for persistent positive PCR results in non-RI cases were attributed to prolonged viral shedding of likely non-viable virus.

The diagnoses of all first episode of infections spanned across April 2020 and May 2021. The diagnoses of all 2nd episode of “possible reinfections” occurred between January and June 2021, which was the time-period in Singapore where the Delta variant emerged in Singapore.

Table 2 shows the differences in clinical, serologic and microbiologic characteristics between non-RI and RI groups. Almost all reported cases were male with an average age of 35 years. Only one person evaluated received two doses of the Pfizer-BioNTech vaccination and was eventually assessed not to have reinfection.

**Table 2:** Characteristics of non-reinfection and confirmed reinfection cases in Singapore

	Not reinfection (n = 42)	95% CI	Confirmed reinfection (n = 32)	95% CI	p-value
Male (%)	39 (93)		32 (100)		
Average age, years (range)	34 (22–51)		35 (26–47)		
Interval between 1st and 2nd episode, days (range)	186 (1-410)	144–228	297 (1-397)	267–327	< 0.001
Ct value of initial PCR at 2nd episode, mean (range)	34 (19–42)	32–36	23 (9–38)	20–26	< 0.001
Repeat PCR performed during 2nd episode (%)	18		17 (53.1)		
Mean interval to repeat PCR, days (range)	2.6 (1–7)		1.9 (1–10)		
Negative results on repeat PCR (%)	10 (56)		2 (12)		
Mean Ct value of positive result on repeat PCR (range)	33 (17–40)	27–40	22 (16–32)	18–25	< 0.001
Vaccinated (%)	1 (2)		0 (0)		
Symptoms at 2nd episode					
Fever (%)	4 (9)		2 (6)		0.09
Acute respiratory symptoms (%)	8 (19)		7 (22)		0.1
Asymptomatic (%)	26 (62)		24 (75)		0.1
Whole genome sequencing performed (%)	5 (12)		26 (81)		
Initial quantitative serology performed at 2nd episode (%)	11 (26)		14 (44)		
Mean cPASS inhibition, % (range)	67 (21–100)	49–85	72 (14–100)	50–95	0.7
Mean Roche S titre, unit (range)	969 (7-8560)	737.2-2674.5	3770 (18-11460)	1131.7-6407.4	0.08
Mean Roche N titre, unit (range)	66 (9-200)	25–106	81 (0.7–265)	25.1-137.4	0.7
Consolidation on CXR at 2nd episode (%)	1 (2)		0 (0)		
Intensive care unit admission at 2nd episode (%)	0 (0)		0 (0)		
Oxygen supplementation required at 2nd episode (%)	0 (0)		0 (0)		

Note:

• T-test or Mann-Whitney U test used to compare means

• Chi-square/Fisher’s exact test used to compare proportions

There was strong statistical evidence that RI cases had a longer interval period between 1st and 2nd episode (mean 297 days; 95% CI 267–327) compared to non-RI cases (mean 186 days; 95% CI 144–228). The Ct value of initial PCR at 2nd episode was also found to be significantly lower in RI cases (mean 23; 95% CI 20–26) compared to non-RI cases (mean 34; 95% CI 32–36).

There was no statistical evidence to support any difference in the proportion of individuals who had fever, acute respiratory symptoms or were asymptomatic between both groups. The mean serologic titres or percentage inhibition on various platforms (Roche and cPASS) did not differ significantly at re-presentation between both groups for individuals for whom results were available. Sequential serology results were available for 11 individuals. Amongst these, eight were classified as RI cases and all these cases had either a rise in anti-S/N titre by at least two-fold after 48 h or an anti-S titre of > 1000 U/ml at initial reading. No one required intensive care unit care or oxygen supplementation and all individuals recovered and were discharged well.

Confirmatory WGS results were available for 31 (42%) individuals and 26 (81.3%) of those with re-infections. Of note, WGS was not technically feasible for samples with high Ct values (e.g. > 30). Delta and beta variants were most commonly identified and provide indication of re-infection as these were not ‘wild-type’ and were not circulating during the time period of the first infection. The Delta and beta variants were also not thought to be endemic locally at the time of evaluation of reinfection.

Amongst the five non-RI cases that had WGS data available, two were identified as B.1.351 (beta), and the other three were B.1.1.7 (alpha), B.1.1.25 and B.1.617.2 (delta). While they were not wild-type SARS-CoV-2, they were still considered to be non-RI as sequential PCR tests in subsequent days turned quickly negative or had high Ct values, not in keeping with the viral kinetics of a true reinfection. Amongst the cases of RI, none of the 26 cases for which WGS data was available identified wild-type SARS-CoV-2.

## Discussion

In this report, we describe a systematic programme used to evaluate possible reinfection cases. A rigorous system of RRT and surveillance enabled prompt detection of possible reinfection cases, allowing a coherent and standardized method of evaluation across different public health institutions. This coordinated approach facilitated prompt public health measures to be taken in the setting of the early phases of a novel coronavirus pandemic, where vaccination was not yet available or just being implemented.

The main findings from the analysis of cases were that RI cases had a longer interval period between 1st and 2nd episodes compared to non-RI cases (p < 0.001). We also observed that the mean Ct value of the initial PCR test performed at 2nd episode was lower in confirmed RI cases compared to non-RI cases (< 0.001). This is also corroborated by previous data from our centre that SARS-CoV-2 was non-culturable from specimens with higher Ct values (> 30) [12].

The time period of this study coincided with the peak of the Delta variant wave in Singapore as well as widespread transmission of infection within migrant worker dormitories [10]. The majority young male study population is reflective of this disproportionately affected population. During the two time periods where all first and second episodes of infection occurred, Singapore reported a cumulative local case count of 61,135 and 28,213 new COVID-19 infection respectively [13]. All but one individual in this study were unvaccinated, meaning no vaccine doses received at all, at the time of analysis, with confirmed reinfection cases comprising < 0.1% of all new cases in Singapore, adding to our understanding of the natural history of COVID-19 in unvaccinated individuals. These findings are consistent with that observed by Peltan et al. who described very low rates of reinfection, albeit in an earlier phase of the pandemic [14].

Our study is limited by the lack of complete viral genotype data due to the lack of routine viral genotyping for all positive tests. Compared to WGS, our algorithm may result in non-differential misclassification. We were also unable to calculate the reinfection rate, as we might do in a cohort study, as we were unable to comprehensively evaluate all individuals who were previously infected but did not get reinfected after exposure. Our study was also performed at a time prior to widespread vaccination, the timing of which, in addition to the specific type of vaccine, and the degree of immune escape afforded by circulating SARS-CoV-2 variants may all affect the probability of reinfection.

## Conclusion

COVID-19 reinfections comprised a small number of all reported cases in Singapore during a surge of Delta variant infections in an early phase of the pandemic. We demonstrate how the use of a combination of microbiologic, serologic and genomic criteria to evaluate possible reinfection cases may be useful and can provide a framework for evaluation that may be modified for future similar situations. In particular, because of the RRT programme in place, we found that reinfections were often evidenced by a repeat PCR positive with a low Ct value (with or without clinical symptoms), accompanied by a ‘boosted’ serologic response (e.g. Anti-S antibody titres), and could be corroborated by WGS which showed a new variant (in this case Delta), compared to prevailing the SARS-CoV-2 strain (wild-type) during the index infection.

As the pandemic surges on into its third year, reinfections remain an enigmatic public health threat, often going unnoticed. If the disease phenotype is mild and does not pose a significant burden on health-systems, attention may not be paid as much to re-infections (such as infections with Omicron and its subvariants). Further study into reinfections will help public health practitioners better understand what the transition into an endemic state with SARS-CoV-2 will look like.

## Data Availability

The datasets generated and/or analysed during the current study are not publicly available in accordance with regulations Sect. 4(1) of the Infectious Diseases Act (Chap. 137) under which this study was approved. Persons who require access to the data from this study should contact the corresponding author.
